# Qualitative analysis of challenges and enablers to providing age friendly hospital care in an Australian health system

**DOI:** 10.1186/s12877-021-02098-w

**Published:** 2021-02-27

**Authors:** Alison M. Mudge, Adrienne Young, Prue McRae, Frederick Graham, Elizabeth Whiting, Ruth E. Hubbard

**Affiliations:** 1 Department of Internal Medicine and Aged Care, Royal Brisbane and Women’s Hospital, Brisbane, Australia; 2 University of Queensland School of Clinical Medicine, Brisbane, Australia; 3 Department of Nutrition and Dietetics, Royal Brisbane and Women’s Hospital, Brisbane, Australia; 4 Department of Internal Medicine, Princess Alexandra Hospital, Brisbane, Australia; 5 Metro North Hospital and Health Services, Brisbane, Australia; 6 Centre for Research in Geriatric Medicine, The University of Queensland, Brisbane, Australia

**Keywords:** Health services, Capacity building, Health workforce, Patient care team, Quality of health care

## Abstract

**Background:**

With ageing global populations, hospitals need to adapt to ensure high quality hospital care for older inpatients. Age friendly hospitals (AFH) aim to establish systems and evidence-based practices which support high quality care for older people, but many of these practices remain poorly implemented. This study aimed to understand barriers and enablers to implementing AFH from the perspective of key stakeholders working within an Australian academic health system.

**Methods:**

In this interpretive phenomenenological study, open-ended interviews were conducted with experienced clinicians, managers, academics and consumer representatives who had peer-recognised interest in improving care of older people in hospital. Initial coding was guided by the Promoting Action on Research Implementation in Health Services (PARIHS) framework. Coding and charting was cross checked by three researchers, and themes validated by an expert reference group. Reporting was guided by COREQ guidelines.

**Results:**

Twenty interviews were completed (8 clinicians, 7 academics, 4 clinical managers, 1 consumer representative). Key elements of AFH were that older people and their families are recognized and valued in care; skilled compassionate staff work in effective teams; and care models and environments support older people across the system. Valuing care of older people underpinned three other key enablers: empowering local leadership, investing in implementation and monitoring, and training and supporting a skilled workforce.

**Conclusions:**

Progress towards AFH will require collaborative action from health system managers, clinicians, consumer representatives, policy makers and academic organisations, and reframing the value of caring for older people in hospital.

## Introduction

Longer life expectancy and expectations of “baby boomer” consumers bring challenges for modern health systems as they adapt to the changing face of patient care. Older people may be vulnerable to hospital-associated complications and poor outcomes because of accumulating frailty and disability [1, 2]. Pioneering models of geriatric care can reduce harm and deliver more efficient, patient-centred care for older people [3]. The principles developed in these models must be expanded beyond small specialist units to all acute care settings caring for older adults to realise the benefits for patients and the healthcare system [4–6].

This challenge has led to the concept of the Age Friendly Hospital (AFH), and several authors have proposed supporting frameworks and practices [7–11] (Table 1). Common principles espoused by these frameworks include senior organisational leadership that addresses institutional ageism; a system that respects older patients’ choices about care and care delivery; staff equipped with geriatric knowledge and skills; evidence-based practices (EBP) to reduce hospital-associated complications such as delirium and falls; a well-designed physical environment to promote function; and improved connections to promote smooth transitions across care settings (Table 1).

**Table 1:** Examples of proposed age friendly hospital (AFH) frameworks, including definitions and key constructs

Definition of AFH	Key constructs of AFH framework
“An age-friendly hospital is a hospital promoting health, dignity and participation of persons of older ages” [8] (Taiwan)	Management policies Communication and services Physical environment Care processes
“Core components of a system-wide, acute care program designed to meet the needs of older adults” [7] (USA)	Guiding principles Leadership Patient- and family-centred approaches Geriatric staff competencies Interdisciplinary resources and processes Aging-sensitive practices Organisational structures Physical environment
“To promote excellence in hospital care for acutely ill older adults through the provision of evidence-based service delivery and patient-family focussed care …” [10] (Canada)	A favourable physical environment Zero tolerance towards ageism at all organisational levels Comprehensive services using principles of the geriatric approach Assistance with appropriate decision-making Fostering links between the acute care hospital and the community
“An evidence-informed framework applied organization-wide to help hospitals achieve better outcomes for frail seniors” [11] (Canada)	Organizational support Emotional and behavioural environment Processes of care Ethics in clinical care and research Physical environment
“An age-friendly health system is based on patients’ goals and values, and on improved outcomes and lower costs of care within the walls of the hospital and beyond” [9] (USA)	Leadership committed to addressing ageism A strategy to identify, coordinate with and support family caregivers A clear process for eliciting patient goals and preferences Clinical staff specifically trained in expert care of older adults Care teams that are high performing and can show measurable results for care of older adults A geriatric care prototype specific to older adults A systematic approach for coordinating care with organizations beyond the walls of the hospital

The Queensland State-wide Older Person’s Health Clinical Network is a multidisciplinary network for clinicians interested in improving the care of older people across Queensland, Australia. In 2016, this group led a state-wide survey assessing age friendly principles in hospital care, adapting a Canadian survey [11]. The self-assessment survey was completed by clinical and executive leaders (e.g. directors of geriatrics, nursing, allied health and facility managers) in 23 hospitals across Queensland, and demonstrated several consistent areas of strength and weakness (Table 2). Weaknesses included poorly coordinated clinical and executive leadership; limited engagement of older consumers; limited geriatric education and training across disciplines; limited recognition and prevention of functional decline and delirium; and poor integration of design principles outside specialist geriatric wards [12]*.*

**Table 2:** Summary of findings from previous state-wide survey of AFH care in Queensland [12]

Key AFH construct	Reported strengths and limitations
Clinical and executive leadership	Developing clinical leadership Limited executive leadership Limited coordination (e.g. planning, monitoring, linkages)
Respected and involved consumers	Established systems for protecting decision making and advance care planning Limited involvement of older person in care planning and feedback
Skilled and compassionate staff	Limited training of hospital staff in care of older people Limited graduate education across all disciplines
Evidence-based assessment and management	Established systems for recognising and preventing pressure injuries, falls, adverse drug reactions and malnutrition Developing systems for integrated assessment, care planning and discharge planning Limited systems for recognising and preventing functional decline and delirium
Connected systems	Established systems for referral to subacute and post-acute care Poor communication between emergency department and residential care facilities
Well-designed physical environments	Developing use of older person friendly design principles in specialist units Limited use at organisational level

The current research aimed to gain an in-depth understanding of why these weaknesses occurred and how they might be addressed, by describing the experience of delivering, supporting and/or improving hospital care for older people. Our objectives were to engage stakeholders with personal, clinical, management and academic experience in hospital care for older people, to articulate a vision for providing age-friendly hospital care, and to clarify challenges and opportunities to progress this vision.

## Methods

The study was conducted in hospitals within two publicly-funded health services in Brisbane and their associated universities, who were partners in a newly-accredited Advanced Health Research and Translation Centre. The project steering committee consisted of clinicians and academics from within these organisations with an interest in hospital care of older people. Steering committee members identified potential key informants within their clinical, consumer representative and academic networks, who in turn identified colleagues as additional participants. Potential participants were known by peers for their interest in improving care of older people, providing informed experiences about challenges and successes. Participants included individuals from a range of backgrounds (clinical care delivery, clinical management, clinical research, teaching and training, and health consumer representative) to construct a multi-faceted understanding of the phenomenon of caring for older people in hospital, aligning with our constructivist worldview, i.e. a knowable world mediated by an individual’s conceptual lens [13]. Participants were purposively selected for maximum variation with respect to position, discipline, setting and experience level, and invited to participate via personalised email from AY. A sample size of 20 participants was pre-specified pragmatically for this time-limited project. Multi-site ethical approval was provided by the Royal Brisbane and Women’s Hospital Human Research Ethics Committee, and participating sites approved conduct of the study through their local research governance processes to ensure the research adhered to national guidelines and local policies.

Interviews were conducted by AY, a postdoctoral researcher with qualitative research experience. She had worked as an allied health professional (AHP) in one participating hospital and was known to three participants. Interviews were undertaken at a time convenient to participants, in a private office space in each participant’s workplace. Each participant was provided with the previously conducted state-wide AFH survey report [12] as a starting point for discussion. The interview began with a verbal and written summary of practice gaps identified within that study (Table 2), inviting the participant to discuss their experiences with care of older people in acute settings related to these gaps. Beyond this opening statement, there were no set interview questions. Probing questions were used if required to elicit further information.

All interviews were digitally recorded using a dictaphone and transcribed verbatim by a professional transcription service, and cross-checked by AY. Field notes were taken during the interview for reference during debriefing sessions with the research team and during coding. Written transcripts were emailed to all participants for member checking; five participants provided clarification.

A hybrid deductive-inductive approach was taken to thematic analysis of interview data using an interpretive phenomenological approach (Fereday & Muir-Cochrane, 2006). In the first instance, a deductive approach used the *Promoting Action on Research Implementation in Health Services* (PARIHS) framework [14, 15] as the analytical framework, to allow consideration of barriers and enablers to AFH care with an implementation focus. The first three steps of analysis were informed by the Framework Method [16]:

1. Familiarisation: review of field notes, audio-recording and transcription, noting initial thoughts and impressions
2. Identifying an analytical framework: pre-defined codes and explanatory notes developed based on PARIHS framework (Table 3).
3. Indexing: first four interviews coded independently by AY and AM, compared for consistency and framework refined with two additional codes generated from the data. Remaining interviews were coded independently by AY using the final framework. Indexing was completed using NVivo for Mac (version 10, QSR International).

**Table 3:** Analytical framework based on the *PARIHS* framework [14, 15]

Codes	Explanatory Notes
**Evidence**	
Clinical experience and local information	Experience of individuals, teams, services that influences decision making; outlook to other service models/initiatives
Patient experience	Patient or family engagement in service delivery or design (formal or informal)
Research	Research evidence (e.g. papers, guidelines, conferences), data sources or processes (e.g. local or state-wide databases, audits), evaluation of service delivery
**Recipients**	
Capability	Knowledge, skills, memory, attention and decision processes, behavioural regulation
Motivation	Social/professional role and identity, values, beliefs about capabilities, optimism, beliefs about consequences, intentions, goals, reinforcement, emotion
**Inner Context (ward and organisation)**	
Adequate resources^a^	Time, personnel, equipment, education materials
Culture	Specific to care of older people and factors influencing this (e.g. teamwork, interdisciplinary care, innovation), ward/service line/organisation level
Experience with change	Past implementation/change experiences: positive/ negative, factors associated with success/ failure
Leadership	Formal and informal leaders, ward/service line/executive, across disciplines
Organisational priorities	Specific to care of older people and factors influencing this (e.g. teamwork, interdisciplinary care, innovation)
Structure	Hospital organisational structure (reporting, governance, service lines), physical structure (physical space, layout of wards/hospital), structure of university courses and curriculum,
Systems and processes	Models of care, processes for screening, referral, discharge planning, communication processes,
**Outer context (health service district, state, national)**	
Environmental (in)stability	Change in political or social environment which may affect older people and/or health care
Incentives and mandates	External funding, restrictions,
Policy drivers, priorities	Broad political agenda relating to older people and/or health care
Inter-organisational networks and relationships	Networks and relationships with community (aged care, GPs, community service providers), universities, other health care organisations
Regulatory frameworks	Health care (i.e. accreditation standards) and university (i.e. competency standards for new graduates)
Society values and expectations^a^	Society perceptions related older people and/or health care

^a^These codes were generated inductively from the interview data

An inductive approach was then taken to identify themes within and across data indexed under each code. AY, AM and RH independently reviewed data indexed at three representative codes and summarised themes. These were compared for consistency and discussed until consensus was achieved. AY and AM independently reviewed and summarised data indexed at the remaining codes, and identified themes within and across codes. Theme summaries, exemplar quotes and five transcripts each were provided to the other four authors for review to ensure that themes and selected quotes adequately represented the data.

Rigour was ensured through discussions within the project steering committee during data collection and analysis, and reporting using COREQ guidelines. Trustworthiness was supported by presenting preliminary themes back to participants via email, and in workshop style at meetings of the State-wide Older Person’s Health Clinical Network and the State-wide General Medical Network (each attended by approximately 40 participants, including clinicians, managers, and consumer representatives), with participants invited to provide feedback.

## Results

We identified 42 potential key informants from eight institutions through initial snowball sampling, and invited 26 of these to participate, using purposive selection to optimise diversity of position, discipline, and workplace within the project resources and timeline. One declined, and five were unable to schedule interviews within the project timeline, so that 20 interviews were completed between October 2016 and February 2017. Participants were mostly female (*n* = 16, 80%), and came from a range of discipline backgrounds as shown in Table 4. Mean duration of interviews was 50 min (SD 16). One participant was an appointed consumer representative, and several other participants discussed their health care experiences as carers of older family members. Participants also drew on their previous experience working in other health services, community settings or other organisations.

**Table 4:** Characteristics of interview participants. Position and workplace were the primary setting identified by the participant; four participants identified themselves as academics and clinicians with joint positions between the university and health service

Characteristic	n (%)
Position	
Clinician	8 (40)
Academic	7 (35)
Manager	4 (20)
Consumer representative	1 (5)
Discipline	
Nurse	10 (50)
Allied health professional	5 (25)
Geriatrician	4 (20)
Consumer representative	1 (5)
Workplace	
Health service 1	10 (50)
Health service 2	5 (25)
University 1	3 (15)
University 2	2 (10)
Gender	
Female	16 (80)
Male	4 (20)

**Fig. 1:**
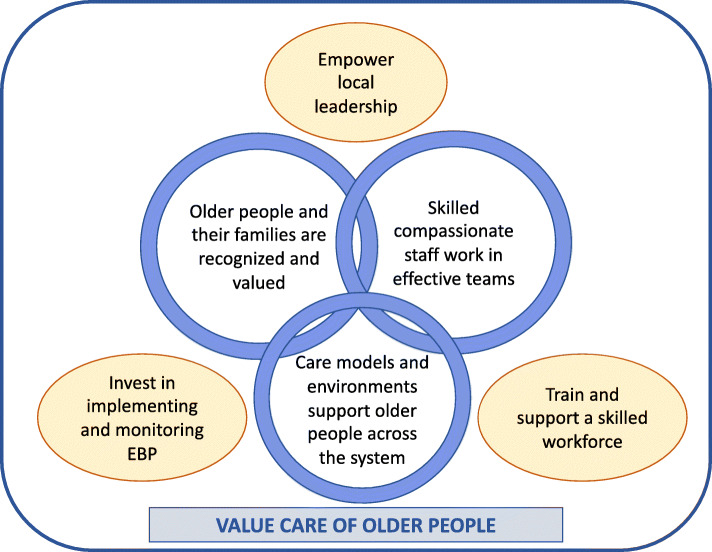
The age friendly hospital (AFH): summary of interview themes. Interlocking circular rings represent the elements of AFH, ovals represent system enablers, and the rectangle represents the foundation value underpinning all system enablers

We identified seven major themes (Fig. 1). Three core elements for achieving systematic, high quality AFH care were:

- Older people and their families are recognised and valued in care
- Skilled compassionate staff work in effective teams
- Care models and environments support older people across the system

Four system enablers were identified to achieve these goals:

- Empower local leadership in older person friendly care
- Train and support a workforce skilled in care of older people
- Invest in implementation and monitoring of evidence-based practices
- Value care of the older person

The latter enabler underpinned all other elements, and required challenging an ageist and efficiency-driven culture.

### Older people and their families are recognised and valued in care

Participants articulated that older patients need to be recognised as a group with particular and often complex care needs requiring specific knowledge and skills, and as legitimate and deserving recipients of acute care. There was a perception that many staff viewed older patients negatively, due to ageism, false expectations set during training, complex care that disrupts and delays hospital processes, and nihilism about outcomes.

“I think there is still a very strong … belief of ‘older people aren’t my real patients. They’re the exception. They’re the difficult ones’” P4, geriatrician, clinician

“We need to generate more positive attitudes to ageing. People say they’re not working with older people, they’re acute care therapists, but that’s who they’ll end up working with” P11, AHP, academic

There was also recognition that older people need to be valued as individuals, with variation in their care needs, expectations, and life experience. Ageist stereotypes contributed to homogenisation of older people, reducing their individual value.

“I think [it’s often said that] ‘older people like this, older people like that’. Older people are actually more individual than you or me. Because over the lifespan, if you think about it, we just keep differentiating, don’t we?” P4, geriatrician, clinician

Some respondents noted that older people may be unable to advocate for their needs, due to generational characteristics or cognitive impairment. Family and carers of older people were recognised as important sources of information, providing advocacy and supporting individualised care. However, many participants reported a transactional approach to engagement e.g. information exchange rather than genuine partnership in decision making and care.

“We certainly had conversations and they were very respectful and polite and all that but it wasn’t shared decision making really.” P12, AHP, clinician (reflecting on consumer experience)

“The carers are often so involved and really good advocates for those people but that’s not what you want when you’re trying to look after a patient … You probably don’t want someone telling you how that person likes their cup of tea or likes to eat their breakfast or take their shower.” P9, nurse, academic

In contrast, some respondents had poignant reflections about their personal experience as care-givers, recognising that family are often invisible, and require substantial confidence and health literacy to initiate engagement.

“It changed once they knew I was a nurse and it made me think ‘What happens to people who don’t have medical knowledge and can’t ask the questions?’ … .Up until then they’d walk into the room and ignore me.” P15, nurse, academic (reflecting on consumer experience)

### Skilled compassionate staff work in effective teams

Knowledge and skills in care of older patients, particularly those with cognitive impairment, were seen as important for all hospital staff.

“Even from the people who work in the ward who are going to handle an older person, who are going to transfer an older person; how are they going to deal with an older person if he has dementia when he needs to move from A to B or he needs to have his meds? So everyone needs to have some sort of training.” P17, geriatrician, academic

Compassion was identified as an important attribute in caring for older people. Experiential learning could enhance compassion, while entrenched ageism, lack of knowledge and mentorship, negative experiences and competing values and demands could erode it.

“I do think that people start with a degree [of compassion] and it can be eroded over time, through experiences of lack of interdisciplinary teamwork, you know, lack of what they see as being important, not being valued.” P3, nurse, manager

“They probably don’t think about it … It’s so easy when you work in that environment all the time. You become immune to what goes on” P9 nurse, clinician

Teamwork was discussed as a critical component of care of older inpatients. Respondents identified value and challenges working in multidisciplinary teams (MDT). Good teamwork provided opportunities for sharing information, prioritising workload, mutual learning and peer support.

“Different people bring the different skill mix to the situation … . I actually love the interaction of the team because it really allows us to talk about the aspects of the older person care in a really MDT approach. We know the view of the doctors, the doctors are able to learn the view of ours and I believe that we learn from each other.” P10, AHP, clinician

However, when multiple team members were involved in care there was a risk of overwhelming patients and families with poorly coordinated interactions. Within teams, professional silos could limit communication of important information, create duplication, and prevent individual providers taking responsibility beyond a limited scope.

“I would hear the nutrition assistant or the dietitian come around to the patient … just saying ‘how’d you go with your [breakfast] this morning’. And I thought, what a missed opportunity, because the nurse was there, you didn’t ask the nurse, you walked straight past them.” P3, nurse, manager

“I think nurses are very quick to go ‘this is not something I know a lot about so I’ll flick it off sooner than I really need to because I know that there’s somebody more expert in that.’” P12, nurse, academic

Important enablers for promoting effective teamwork included face-to-face meetings, working together over time to develop trust and shared values, and open discussion of role boundaries.

“[These medical staff] don’t have any rapport with the allied health team... in comparison to teams that might meet three time a week so you’ve got a bit of rapport. You get to understand how they work, and they get to understand how you work … I think with teams that don’t meet and don’t really know each other, that’s really difficult.” P1, AHP, clinician

### Care models and environments support older people across the system

There was broad recognition that improving care practices required a systematic and system-wide approach. This was challenged by existing professional and governance structures, which are based around “organs”, disciplines and acuity, leading to siloed practices and disjointed systems within and beyond the hospital.

“Why is it that cardiology isn’t also a good place for an older person and why is it age that determines whether you go to cardiology or not?” P4, geriatrician, clinician

“This team comes and looks at X, and this team comes and looks at Y, but how are we all sort of pulling it together?” P14, nurse, academic

Respondents recognised the need for better relationships and communication beyond the hospital, particularly with residential aged care. Respondents admitted to poor mutual understanding, leading to assumptions and conflict in transitions between providers.

“People in both sectors are throwing their hands up in the air, saying “we’re just not being told what we need to be told.”” P11, AHP, academic

There was tension between care of older people being a specialty versus being usual care, including some tension between geriatricians and general physicians. Staff with specialist skills could provide mentorship and support for generalist staff, but could also increase complexity, and the concept of specialist care models was challenged by the large number of older patients.

“Maybe all older people need a geriatrician just to look at, to watch over them, not necessarily be the primary carer but to actually have input into their care.” P2, nurse, manager

“Sometimes [emergency department outreach service] is involved, [community interface service] is involved, the geriatrician’s involved and throw in the Older Persons’ Psychiatrist as well and then there’s a lot of varying opinions sometimes. I think we need to look at who is owning the patient.” P19, geriatrician, clinician

Respondents valued comprehensive screening and assessment for older inpatients, but recognised that screening could be inconsistent, and did not always translate into care practices. Reasons included professional boundaries and expectations, limited support systems, and balancing time spent between screening and delivering actions.

“Even the new care plans that we’ve got in place, it talks about cognition but it’s not clear. It’s just another ticked box. It refers to using the CAM [Confusion Assessment Method], but then they don’t have the CAM and they don’t have that training.” P20, nurse, clinician

“Most of the time, you do the assessment, you do the discharge planning, and there’s so little time left for the really proper targeted goal-oriented therapy” P10, AHP, clinician

There was a developing awareness of evidence-based environmental design features for older people, particularly by respondents with experience in residential aged care and specialist older person’s wards. However, staff were often circumspect about their influence over decision makers, and whether the principles would be realised in practice.

“We don’t actually think about what we do in these hospitals. We think about colour combinations as being something that is pleasant, whereas it’s not necessarily that functional from an older person point of view.” P2, nurse, manager

“We are fighting to get that ACE [Acute Care of Elders] ward … It is being constructed, but it’s the colour code and the dining area and the toilet design … Hopefully it’s going to be the way we have planned.” P5, geriatrician, clinician

### Empower local leadership in older person friendly care

There was a recognised need for multi-level leadership to provide strategic and practical support for older person friendly practices. However, there was a lack of confidence in executive leadership and accountability, and challenges with governance structures which support existing organisational silos.

“I think it’s in the back of everyone’s mind but no-one’s had the ability, if you like, to move it forward.” P6, nurse, manager

“We’re going to have to have a new way of thinking about how we structure our governance. Even though I might have a service line to look after, my remit for the older person might have to be across the organisation and if that’s the case then the other services have to be prepared for me to paddle in their place a little bit.” P2, nurse, manager

As a result, change and advocacy were driven predominantly by passionate informal leaders, limited by time constraints of their ‘real job’ and their individual capacity to galvanise action. Poor role recognition and slow progress often led to frustration, while establishing alliances across traditional discipline and department boundaries helped gain momentum. There was also strong reliance on local champions, particularly nurse unit managers. They provided local leadership through motivating staff, setting expectations, coaching and modelling, but required continuing support.

“Unfortunately he’s a one-man band … This is a huge organisation and as enthusiastic as one person is, there are still real limits around how quickly you can start.” P12, nurse, academic

“I would go to geriatric things and come back all keen and try to get basic age friendly principles into place, basic signage, and I had no traction.” P20, nurse, clinician

### Train and support a workforce skilled in care of older people

Respondents articulated the need for both a formal gerontology curriculum and opportunities for interacting with older people from early undergraduate years. Limited undergraduate training in care of older people was recognised across all professions, attributed to low priority by teaching institutions. Gerontology content was generally delivered as elective rather than core curriculum, clinical placements were limited, and there were minimal opportunities for post-graduate study in gerontology.

“Part of the challenge in preparing a workforce is that you need to develop [teamwork] skills just as much as the development of the technical and know-how skills that fascinate accrediting authorities but actually don’t really serve people well in terms of being work-ready.” P18, AHP, academic

Consequently, most training was occurring in the workplace. The importance of experiential learning was well articulated, but exposure to training was generally ad hoc, depending on early placements on geriatric wards or exposure to passionate champions. There was a recognised lack of skilled teachers and mentors with dedicated time to provide training and support, and reliance on individually-sought external conferences and courses.

“If you don’t go through a geriatric unit or work with a geriatrician who is maybe working as a general physician, other than that, I don’t think they will get formal geriatric training.” P5, geriatrician, clinician

“It’s just where you end up working. You’re provided with on-the-job training … There’s lots of external courses and conferences but there’s nothing specific here.” P6, nurse, manager

There were challenges in delivering hospital-based education including multi-level learning needs, competing mandatory training, limited education time, high staff numbers and turnover, and the limited value of traditional ‘in-service’ approaches for complex education and skills training. External agencies provided some educational opportunities, but there was a recognised need for continuing support and ‘hands-on’ training, particularly in caring for people with cognitive impairment.

“If the skill mix is poor and you haven’t got those other people with experience, then how do you actually get that?” P13, nurse, clinician

“It’s educating staff … coaching them and mentoring them is the difference. It’s constant follow-up. All this in-service and interest groups and stuff like that, it’s great. It’s not enough. It’s not enough to create change and keep motivating people.” P16, nurse, clinician

### Invest in implementation and monitoring of evidence-based practices

Evidence-based practices (EBP) to achieve better outcomes for older people were well recognised, but poorly translated into practice. Barriers included lack of staff knowledge, lack of prioritisation by teaching and service organisations, resistance to change, and lack of investment in implementation and change management methods.

“It was interesting hearing all the research out there and all the money that’s been invested into research. It stops there and that’s the thing. There is so much stuff out there saying how we should be doing things. Then the next step is investing into implementing into practice.” P16, nurse, clinician

Translational efforts included credible experts from other organisations, local adaptation of guidelines and standardised orders, education and mentoring, and local data to prioritise issues and highlight gaps in EBP. These efforts were driven largely by informal leaders. Progress required champions willing to change practice, and continuing support and monitoring within the local context.

“I think in [ward name] the model works better because they’ve got such passionate and enthusiastic staff so they’re actually wanting to change the culture of how they care for older people.” P19, geriatrician, clinician

“I think people [staff] will fall unless we really monitor, I think they’ll fall back to their old pattern.” P2, nurse, manager

Although several respondents appeared confident that organisational audits, incident monitoring and benchmarking processes provided appropriate data for monitoring care and outcomes of older patients, others were sceptical about data quality and use, and identified a lack of age-stratified reporting. They articulated that investment in targeted measurement was essential, needing staff with skills and dedicated time for reliable data collection and interpretation.

“They’d tell you how wonderful all their data is and how they collect all this data. To me it’s all smoke and mirrors. It’s a bit like quality and safety data … there’s not a lot of rigour in how it’s collected.” P12, nurse, academic

### Value care of older people

Most respondents believed that staffing and resourcing to support care of older people was inadequate, because it was a low organisational priority. The powerful competing demand of throughput, with an emphasis on emergency department wait times, length of stay and patient discharge, was a major disincentive to person-centred and quality-focussed resource allocation. At the same time, care for older people was reported as more difficult and complex, requiring more resources and disrupting task-driven care pathways. This created challenges articulating the value proposition for investing in care of older people.

“Everything’s about NEAT [National Emergency Access Targets]. They don’t realise that it’s actually what happens in between that effects NEAT” P19, geriatrician, clinician

“The problem is that [geriatric interventions] are often quite expensive and how do we make them cost effective and show our bean counters that they’re actually of value?” P6, nurse, manager

Many respondents felt they were constantly arguing for resources for older patients, which were often secured in an ad hoc manner. Participants attributed successes to networking with other champions to pool resources and energy, and alignment with accreditation standards or other strategic policy. The consumer representative highlighted the under-utilised influence of politically aware older consumers.

“[Older consumers] are the guys that have the money, we’ve got the influence, and there’s lots of us, and we’re just starting to wake up to the fact that we’ve got to start making things a lot better for ourselves.” P7, consumer representative

## Discussion

Using in-depth interviews with key informants recognised by peers for their interest and involvement in age-friendly hospital care, we propose an action-focussed model for hospitals to improve how we care for older people (Fig. 1). This model emphasises intersections between how older patients and families are seen and involved in care, how staff are trained and connected, and how the care environment is created. Our respondents highlighted substantial challenges and opportunities in leadership, workforce development, and implementation of EBP required to support these elements, and combat an ageist and efficiency-driven culture.

The central importance of patient and family involvement supports and extends previous AFH frameworks (Table 1). Engagement must go beyond eliciting patient preferences and sharing information with families [9, 10], to valuing older patients and their families as legitimate partners in care decisions and provision [17]. This is congruent with a systematic review of acute care experiences from the perspective of hospitalised older people and their carers [18], which concluded that genuine engagement requires recognition, reciprocity, and involvement.

Previous frameworks have recognised the need for staff geriatric competencies, but our study indicates that compassion and teamwork are essential to complement geriatric knowledge, and require experiential learning. Consistent with other reports [19, 20], our respondents reported lack of dedicated gerontology teaching time, and lack of expert teachers and mentors in undergraduate and graduate training across all disciplines. They identified that traditional teaching methods must be supported by opportunities for interdisciplinary training, and positive mentored experiences to demonstrate skills and values in practice. Creating such opportunities will require leadership [11] and cooperation between education and health care sectors to prioritise gerontology curriculum and support effective mentors.

Respondents recognised the central importance of geriatric care principles (e.g. comprehensive risk assessment linked to integrated multi-disciplinary care planning) and how teamwork and the physical care environment could enhance or challenge delivery of this evidence-based care [7, 10]. Many respondents were frustrated by perceived failure to translate EBP into everyday practice. This challenge has been recognised internationally, with poor uptake of effective demonstration models into other settings [3]. Effective implementation of EBP for older people requires leadership support [21] but our findings illustrate the complexity of providing leadership in a system traditionally organised along disciplines and organ-based specialties. This leads to reliance on motivated individuals forced to build their own skills and networks. Strategic collaborations which empower and connect these individuals, encourage cross-disciplinary partnership, create legitimate capacity for role modelling for other staff, and advocate for investment in implementation of EBP could enhance organisational capability in care of older people. Robust systems for measuring and monitoring age-stratified outcomes would support visibility and facilitate improvements [7].

Our three key enablers of leadership, training and investment (Fig. 1) are similar to the concepts of authority, awareness and resources identified in a recent review of successful dementia-friendly hospital practices [22]. Clearly identifying the value of caring for older people in hospital is essential to activate these enablers, but remains challenging in a context which prioritises efficiency and throughput [23]. In a time-driven system, staff can feel helpless and frustrated by complex care needs (e.g. due to cognitive impairment or disease complexity), and older patients can become isolated and ignored. Powerful social discourses about ageing support this behaviour [6]. Our respondents recognised that these additional needs could not be convincingly framed within the prevailing efficiency paradigm, and that progress requires aligning with other powerful policy incentives such as quality standards and consumer expectations. Careful selection of communication strategies is critical to ensure thoughtful discourse and avoid perpetuating ageist and negative stereotypes of health system “burden” rather than recognition of legitimate and specific healthcare needs [24, 25].

Our study deliberately selected participants who were knowledgeable advocates for older person care, and we acknowledge that they may not recognise barriers and enablers experienced by other staff and consumers. However, many of our findings are congruent with barriers identified in other studies exploring the perspectives of older acute care consumers, their carers and staff. A systematic review of qualitative studies of older patients’ experiences of acute care found that the dominant patient narrative concerns the relational aspects of care, highlighting the central importance of recognising and valuing older people [18]. Patients and their families expected their values and perspectives to be respected, and wished to feel welcome and cared for, and to receive sufficient appropriate information to participate in decisions and maintain a sense of control. Delivering these essential features of dignified care requires skill, self-awareness and compassion to allow effective listening and learning from older patients [26]. An ethnographic study in Canada, which included older patient and carer interviews, staff interviews and direct observations of care, highlighted the challenges of poorly designed environments, bureaucratic demands focussed on efficiency, and a chaotic and frenetic social environment which compromised delivery of older person-centred care, especially to those requiring additional time for their care [23]. This poor fit could leave patients and carers feeling isolated and staff feeling dissatisfied and frustrated, perpetuating ageist stereotypes through negative staff attitudes. A similar ethnographic study in the United Kingdom found that almost all staff believed that acute hospital wards were ‘not the right place’ for older people, despite being the main occupants [27]. Geriatric training was limited, and the priorities of organisational throughput and risk averseness conflicted with dignified person-centred care, leading to disempowering environments and care practices. In order to support staff to deliver person-centred care, the authors recommended key messages to counter ageist attitudes, greater health professional education in geriatrics, involving consumers in environmental redesign, broader measures of quality of care, and support for ward leaders [27].

The practical enablers of AFH proposed from our findings (Fig. 1) are being demonstrated in large scale AFH initiatives internationally. For example, based on a system-wide analysis of AFH practices [11], the Senior-Friendly Hospitals initiative in Canada created action communities for clinical and executive champions from hospitals throughout Ontario. They invested in intensive skill development in geriatrics, quality improvement and change leadership to promote rapid uptake of evidence-based approaches to delirium and functional decline. The expanded Senior-Friendly Care program now also includes self-assessment and training materials and implementation support [17]. Similarly, the large Age-Friendly Health System initiative in the United States of America [28] has invested in supporting local hospital and clinical leaders to self-assess, implement and monitor key evidence-based care processes identified as “the 4 M’s” (mentation, mobility, medications and ‘what matters to me’). Participants are supported to lead an interprofessional team to adapt existing care models and environments in collaboration with older consumers. In both these models, high level endorsement and practical support by credible organisations promotes the value of caring for older people, and participation in a community of practice empowers local leaders to lead and monitor evidence-based practice, and share resources to enhance workforce knowledge and skills.

Strengths of our study include a multidisciplinary steering group and diverse respondents from varied setting and disciplines. Informants engaged enthusiastically and provided a comprehensive view from multiple perspectives. Internal and external validity were supported by several researchers coding and checking themes, member checking with participants, and discussion with expert groups from inside and outside the participating organisations. Our hybrid approach using a recognised implementation framework helped us move beyond descriptions to actionable themes to inform improvement. We also recognise some limitations. Despite a diversity of disciplinary and practice backgrounds, most participants were female. The stimulus (survey report) provided to frame interviews, and snowball methods of recruitment, mean that participants may have been aware of the perspectives of the research team, which may have influenced their responses. Pragmatic constraints on sample size mean we cannot be certain that data saturation was reached, although consistent themes were voiced across diverse settings and disciplines. Our settings were inner and outer metropolitan hospitals within two publicly funded health services in Australia, and our findings are likely to be influenced by this cultural context. We did not sample policy makers, which may have under-represented codes related to the outer setting. We only had one consumer representative participant, and further research exploring the consumer viewpoint would provide valuable additional information.

## Conclusions

Our findings inform actionable opportunities for clinicians, managers, consumer representatives, academics and policy makers to work together to create AFH. Universities and training institutions should encourage access to gerontology curricula for all disciplines, and collaborate with health services to provide mentored training experiences and practice teamwork skills. Clinicians and consumer representatives must advocate for investment in age-friendly education and training, role model respectful and dignified care and teamwork, and actively listen and learn from older consumers and carers to improve care systems and environments [25, 26, 29]. Hospital managers should recognise and resource local champions as key resources for training and translation, and invest in adequately resourced interdisciplinary teams. They should support evidence-based age-friendly care practices and environments, with age-stratified measurement of appropriate outcomes to monitor effectiveness [26]. Policy makers must recognise that prioritising efficiency can create perverse cultural incentives with serious consequences for vulnerable older patients, and provide safeguard mechanisms such as age-friendly quality and experience measures [30]. Researchers can work collaboratively with all stakeholders to identify effective leadership structures for AFH, implement EBP at scale, and validate reliable, feasible and responsive measures of care, experience and outcomes that matter to older patients. Multi-level collaborative leadership is required to challenge ageism and truly value older people if we are to redesign our hospital to provide age-friendly and dignified care [26].

## Data Availability

The datasets generated and/or analysed during the current study are not publicly available according to the institutional ethics approval granted, but are available from the corresponding author on reasonable request.
